# Development and validation of interpretable machine learning models for predicting stroke in NVAF patients with CHA_2_DS_2_-VA scores ≤1

**DOI:** 10.3389/fcvm.2026.1725168

**Published:** 2026-04-28

**Authors:** Tanggang Gao, Xiaomin Zhang, Tianyi Ma, Meijuan Li, Bingbo Hou, Ze Lin, Jiayi Cai, Sen Huang, Xiping Liu, Yanan Zhao, Siyang Ji, Weibing Guo, Wenxin Xue, Feng Gao

**Affiliations:** 1 Department of Cardiology, Zhongshan Hospital, Xiamen University, Xiamen, Fujian, China; 2 Department of Endocrinology, West China Xiamen Hospital of Sichuan University, Xiamen, Fujian, China; 3 The School of Clinical Medicine, Fujian Medical University, Fuzhou, Fujian, China; 4 Department of Cardiology, Xiamen Haicang Hospital, Xiamen, Fujian, China

**Keywords:** Asian population, biomarkers, CHA₂DS₂-VA, machine learning, non-valvular atrial fibrillation, SHAP, stroke risk prediction

## Abstract

**Background:**

Non-valvular atrial fibrillation (NVAF) patients with CHA₂DS₂-VA ≤1 face uncertainty in stroke risk assessment, particularly in Asian populations. Machine-learning (ML) models offer improved accuracy for individualized risk prediction.

**Methods:**

This single-center, retrospective study at Zhongshan Hospital, Xiamen University (January 2022–January 2025) included 82 NVAF-related stroke cases and 164 matched non-stroke controls with CHA₂DS₂-VA ≤1. Data encompassed demographics, comorbidities, laboratory markers, and echocardiographic parameters. Following stratified train-test split (80%:20%), feature selection used univariable logistic regression and least absolute shrinkage and selection operator (LASSO) regression, retaining the intersection of variables selected by both methods. Three nested predictor sets were defined: Model A (clinical and routine laboratory variables), Model B (Model A plus Cardiac biomarkers), and Model C (Model B plus echocardiographic parameters). ML algorithms [logistic regression (LR), random forest (RF), XGBoost (XGB)] underwent nested cross-validation for hyperparameter tuning. Performance was evaluated by area under the receiver operating characteristic curve (AUC), sensitivity, specificity, precision, and F1 score. Shapley additive explanations (SHAP) were applied to the best-performing ML algorithm in the test set to evaluate the contributions of individual features.

**Results:**

Stroke cases were older, with higher E/e′ ratio, and elevated N-terminal pro-B-type natriuretic peptide (NT-proBNP), C-reactive protein (CRP), and white blood cell count (all *P* ≤ 0.01). Comorbidities such as heart failure, hypertension, and age 65–74 years were more prevalent in stroke cases (all *P* ≤ 0.05). Feature selection yielded seven predictors: age, CRP, E/e′ ratio, left ventricular ejection fraction (LVEF), NT-proBNP, triglycerides, and white blood cell count. In the training set, XGB employing Model C achieved an AUC of 0.905 (95% CI: 0.877–0.933). In the test set, XGB employing Model C yielded the AUC (0.906; 95% CI: 0.826–0.985). SHAP analysis identified NT-proBNP as the most influential feature, with elevated NT-proBNP and E/e′ levels associated with increased predicted risk and higher LVEF linked to decreased risk.

**Conclusions:**

ML models incorporating cardiac biomarkers and echocardiographic parameters improve stroke risk stratification in low- to moderate-risk NVAF patients, supporting personalized anticoagulation strategies.

## Introduction

Non-valvular atrial fibrillation (NVAF) is one of the most common arrhythmias in clinical practice and is strongly associated with an increased risk of ischemic stroke, leading to substantial morbidity and mortality (1, 2).In this study, non-valvular atrial fibrillation refers to atrial fibrillation in the absence of moderate-to-severe mitral stenosis or mechanical heart valves, a distinction that remains relevant for clinical research and risk stratification. Current European Society of Cardiology (ESC) guidelines recommend the CHA₂DS₂-VA score for stroke risk stratification in patients with NVAF, which is a simplified version of the CHA₂DS₂-VASc score that excludes female sex as a risk factor (3).

According to the ESC guidelines, anticoagulation is a Class IIa recommendation for patients with a CHA₂DS₂-VA score of 1, due to the borderline annual stroke risk. However, the clinical decision to initiate anticoagulation in this population remains controversial. Notably, several studies have shown that even NVAF patients classified as “ low-to-moderate-risk” (CHA₂DS₂-VASc ≤1) may still experience a non-negligible risk of stroke, particularly in Asian populations, where the incidence ranges from 0.78% to 3.50%, significantly higher than in Western cohorts (0.50%–1.55%) (4–11).

The CHA₂DS₂-VA score, based solely on clinical history, may overlook important mechanistic and pathophysiological contributors to thromboembolism, such as atrial cardiomyopathy, hemodynamic changes, and hypercoagulability (12). In recent years, artificial intelligence has been increasingly applied to atrial fibrillation management, including diagnosis, treatment, and prediction of clinical outcomes (13–16). Compared with traditional approaches, machine learning (ML) excels at analyzing complex, nonlinear relationships among diverse clinical, demographic, and laboratory variables, thereby enabling more precise risk stratification. Additionally, ML algorithms can adaptively incorporate new data to facilitate real-time prediction updates. Nishi et al. developed a gradient boosting tree model to predict adverse clinical events in patients with NVAF (15). However, their study provided limited insights into the contributions of individual variables, which constrained clinical interpretability. To date, ML applications in low-to-moderate-risk NVAF patients—particularly those with CHA₂DS₂-VA scores ≤1—remain limited, with scant emphasis on identifying key risk factors.

Patients with CHA₂DS₂-VA scores ≤1 represent a clinically important subgroup in whom anticoagulation decisions remain uncertain. Although traditionally classified as low risk, accumulating evidence indicates that a subset of these patients may still experience thromboembolic events and may harbor additional risk factors not captured by conventional clinical scores (17, 18). Therefore, improved risk stratification in this population is of clear clinical importance.

To address this gap, we conducted a retrospective case-control study in NVAF patients with CHA₂DS₂-VA scores of 0–1. Our aim was to identify additional risk factors beyond conventional clinical scores and to develop an interpretable machine-learning model for more individualized stroke risk stratification in this clinically challenging population.

## Methods

### Study population

This single-center, retrospective, observational study was conducted at Zhongshan Hospital, Xiamen University, from January 2022 to January 2025. We enrolled hospitalized patients with NVAF who experienced NVAF-related stroke, confirmed by neuroimaging (computed tomography or magnetic resonance imaging) and clinical evaluation. Cardioembolic stroke was defined based on an embolic clinical–imaging pattern combined with a plausible cardiac embolic source, such as atrial fibrillation or left atrial appendage thrombus, identified through rhythm monitoring and echocardiography (19). Diagnoses of comorbidities followed recent guidelines or expert consensus. The inclusion criteria were a confirmed diagnosis of NVAF. The CHA₂DS₂-VA score incorporates the following components: chronic heart failure (1 point), hypertension (1 point), age ≥75 years (2 points), diabetes mellitus (1 point), prior stroke, transient ischemic attack (TIA), or arterial thromboembolism (2 points), vascular disease (1 point), and age 65–74 years (1 point) (3). Patients were excluded if they had a CHA₂DS₂-VA score ≥2, moderate-to-severe mitral stenosis, a history of mechanical heart valve replacement, a patent foramen ovale, or missing values in key clinical or laboratory variables, which precluded inclusion in the final analytic cohort. Controls were selected by stratified random sampling. Among NVAF patients with CHA₂DS₂-VA ≤1 and no history of stroke, strata were defined by sex and month of enrollment. For each case, two controls were randomly sampled without replacement from the same stratum, yielding a 1:2 case-to-control ratio.

### Data collection

Baseline clinical data were abstracted at index admission from the electronic medical record, including demographics (age, sex, smoking status, alcohol use) and comorbidities (chronic heart failure, hypertension, diabetes, vascular disease). Laboratory data were collected from the initial blood draw at presentation. Because pre-stroke laboratory values were unavailable, admission results were used consistently, and the earliest measurement obtained was recorded in accordance with the standardized protocol. Echocardiographic parameters, including left atrial diameter (LAD), left ventricular ejection fraction (LVEF), and the E/e′ ratio (early diastolic transmitral flow velocity to early diastolic mitral annular tissue velocity), were extracted from the first transthoracic echocardiogram performed during the index admission. Features with >20% missing values and samples with >20% missing features were excluded.

### Train–test split and leakage control

The dataset was stratified by outcome and randomly split into training (80%) and test (20%) sets to preserve outcome distribution. All preprocessing steps, including imputation, transformation, removal of highly correlated features, and feature selection, were performed solely on the training set. Multiple imputation by chained equations (MICE) was applied with three imputed datasets (m = 3); variable selection was conducted independently for each dataset, with the final feature set determined by majority vote (selection frequency ≥2/3). The preprocessing pipeline was then applied to the test set without refitting to prevent information leakage.

### Data preprocessing, correlation pruning, and feature selection

Following the stratified train–test split, collinearity analysis was performed on the imputed training dataset. Pairwise Spearman correlations were computed among continuous predictors, and pairs with |ρ| ≥ 0.80 were pruned by retaining the more clinically relevant variable; only retained variables advanced to feature selection. All screening was confined to the training set. Two complementary feature selection methods were applied. Method A used univariable logistic regression with a significance threshold of *P* < 0.05. Method B employed LASSO regression within each of the three imputed training datasets. For each dataset, a standardized model matrix was constructed, 5-fold cross-validated LASSO was performed, and variables selected at *λ*.1se were recorded. Features selected in ≥2/3 of imputed datasets were retained. The final feature set for machine learning modeling was defined as the intersection of features from Methods A and B. Three nested predictor sets were defined for downstream modeling: Model A (clinical and routine laboratory variables, excluding cardiac biomarkers and echocardiographic parameters), Model B (Model A plus cardiac biomarkers), and Model C (Model B plus echocardiographic parameters).

### Model development and hyperparameter tuning

For each predictor set, three algorithms were trained: multivariable logistic regression (LR), random forest (RF), and extreme gradient boosting (XGB). Model selection employed nested cross-validation (CV) on the training set, with an outer 5-fold CV repeated three times and an inner 3-fold CV for hyperparameter tuning. All preprocessing steps were integrated into the modeling pipeline. Predictions from the outer validation folds were aggregated to obtain robust performance estimates.

### Performance evaluation and visualization

Model discrimination was evaluated by the area under the receiver operating characteristic curve (AUC), with 95% confidence intervals (CI) derived using DeLong's method. Sensitivity, specificity, precision, and F1 score were computed at the threshold determined by Youden's index. These metrics were calculated for each ML algorithm (logistic regression, random forest, extreme gradient boosting) across all predictor sets (Model A, Model B, Model C). Following model selection, each algorithm was refitted with optimal hyperparameters on the entire training set and evaluated on the test set.

### Model explainability

To address the black-box nature of machine learning models, Shapley Additive Explanations (SHAP) values were computed for the best-performing ML algorithm, selected based on the highest test set AUC, using the test set to ensure unbiased estimates. TreeSHAP was applied for XGB, while model-agnostic SHAP was used for LR and RF. Global importance and beeswarm summary plots were generated to illustrate feature contributions and their impact on predictions.

### Statistical analysis

Normality of variables was assessed using the Shapiro–Wilk test. Continuous variables were reported as mean ± standard deviation (SD) for normally distributed data or as median [interquartile range, IQR] for skewed data, with between-group comparisons performed using the two-sample t test or Wilcoxon rank-sum test, as appropriate. Categorical variables were summarized as counts (%) and compared using the *χ*^2^ test or Fisher's exact test for expected counts <5. All analyses were conducted in R 4.5.1 (R Foundation for Statistical Computing, Vienna, Austria) using packages including tidyverse, recipes, rsample, glmnet, ranger, xgboost, pROC, mice, fastshap, and shapviz. A fixed random seed was set to ensure reproducibility. All statistical tests were two-sided, with *P* < 0.05 considered significant.

### Ethics

The study protocol complied with the 1975 Declaration of Helsinki and was approved by the Ethics Committee of Zhongshan Hospital, Xiamen University.

## Results

### Characteristics of the study cohort

**Figure 1:**
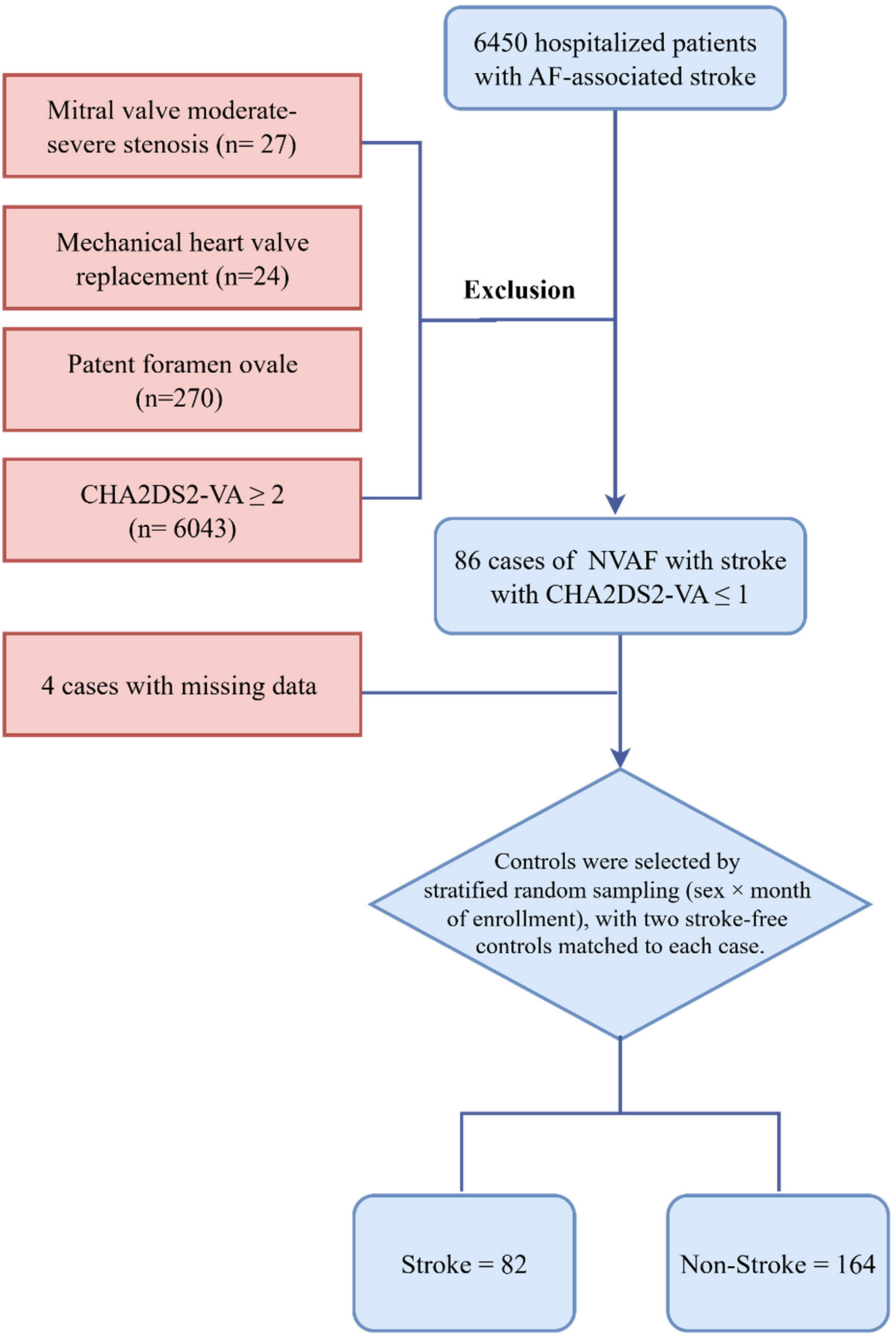
The flow chart of study cohort selection. A total of 86 patients with non-valvular atrial fibrillation (NVAF) and CHA₂DS₂-VA ≤1 were identified, among whom 82 were included in the final analysis. Controls (*n* = 164) were selected by stratified random sampling, matched 2:1 by sex and month of enrollment.

From January 2022 to January 2025, 6,450 inpatients with NVAF-related stroke were screened. Patients with a CHA₂DS₂-VA score ≥2 (*n* = 6,043), moderate-to-severe mitral stenosis (*n* = 27), prior mechanical heart valve replacement (*n* = 24), or patent foramen ovale (*n* = 270) were excluded, resulting in 86 patients with a CHA₂DS₂-VA score ≤1 at the time of stroke. Four patients were excluded for missing key clinical or laboratory data, yielding 82 stroke patients for analysis. Additionally, 164 non-stroke controls were selected via stratified random sampling (by sex and month of enrollment) to achieve a 1:2 case-to-control ratio. The cohort assembly is shown in Figure 1.

Compared with the non-stroke group, the stroke group was older [59.50 (55.00, 64.75) vs. 52.00 (46.75, 62.00) years, *P* < 0.001], with larger left atrial diameter [LAD; 42.00 (39.00, 46.00) vs. 39.00 (35.00, 43.00) mm, *P* = 0.001] and higher E/e′ ratios [11.90 (10.40, 14.10) vs. 9.60 (7.68, 11.07), *P* < 0.001]. Levels of high-sensitivity cardiac troponin T [hs-cTnT; 11.05 (8.33, 15.47) vs. 6.95 (4.97, 9.62) ng/L, *P* < 0.001], N-terminal pro-B-type natriuretic peptide [NT-proBNP; 813.70 (507.75, 1,114.93) vs. 182.00 (75.22, 412.00) ng/L, *P* < 0.001], C-reactive protein [CRP; 5.40 (2.12, 10.35) vs. 1.70 (0.80, 3.50)], white blood cell (WBC) count [8.05 (6.50, 9.78) vs. 6.90 (6.00, 8.12)] and D-dimer [0.62 (0.22, 1.50) vs. 0.30 (0.20, 0.52)] were significantly higher in the stroke group (all *P* < 0.001). Low-density lipoprotein cholesterol (LDL-C) was higher, whereas blood urea nitrogen (BUN) and serum creatinine (Scr) were lower (all *P* ≤ 0.01). Heart failure (11.0% vs. 2.4%, *P* = 0.012), hypertension (31.7% vs. 18.3%, *P* = 0.028), and age in the 65–74-year range (25.6% vs. 14.0%, *P* = 0.040) were more prevalent in the stroke group, with a higher proportion having CHA₂DS₂-VA scores of 1 (76.8% vs. 47.6%, *P* < 0.001). No significant differences were observed in sex distribution, smoking, alcohol use, vascular disease, atrial fibrillation type, international normalized ratio (INR), fibrinogen, hemoglobin, platelet count, uric acid, or glucose levels (Table 1).

**Table 1:** Baseline characteristics.

Variable	Total (*n* = 246)	Non stroke (*n* = 164)	Stroke (*n* = 82)	*P* value
Male (%)	162 (65.9%)	108 (65.9%)	54 (65.9%)	1.00
Smoke (%)	110 (44.7%)	68 (41.5%)	42 (51.2%)	0.189
Alcohol (%)	51 (20.7%)	33 (20.1%)	18 (22.0%)	0.868
HF (%)	13 (5.3%)	4 (2.4%)	9 (11.0%)	0.012
Diabetes mellitus (%)	8 (3.3%)	5 (3.0%)	3 (3.7%)	1.00
Hypertension (%)	56 (22.8%)	30 (18.3%)	26 (31.7%)	0.028
Vascular disease (%)	20 (8.1%)	16 (9.8%)	4 (4.9%)	0.284
Age65–74 (%)	44 (17.9%)	23 (14. 0%)	21 (25.6%)	0.040
CHA2DS2-VA Score				<0.001
0	105 (42.7%)	86 (52.4%)	19 (23.2%)	
1	141 (57.3%)	78 (47.6%)	63 (76.8%)	
AF type				0.125
Paroxysmal AF (%)	153 (62.2%)	108 (65.9%)	45 (54.9%)	
Non-paroxysmal AF (%)	93 (37.8%)	56 (34.1%)	37 (45.1%)	
Age (years)	57.00 [48.25, 63.00]	52.00 [46.75, 62.00]	59.50 [55.00, 64.75]	<0.001
LAD (mm)	40.00 [36.00, 44.00]	39.00 [35.00, 43.00]	42.00 [39.00, 46.00]	0.001
E/e′	10.32 [8.21, 12.35]	9.60 [7.68, 11.07]	11.90 [10.40, 14.10]	<0.001
LVEF (%)	65.00 [59.10, 68.00]	66.00 [62.00, 69.25]	61.00 [55.25, 65.30]	<0.001
hs-cTnT (ng/L)	8.15 [5.50, 11.50]	6.95 [4.97, 9.62]	11.05 [8.33, 15.47]	<0.001
NT-proBNP (ng/L)	314.61 [110.00, 735.89]	182.00 [75.22, 412.00]	813.70 [507.75, 1,114.93]	<0.001
INR	1.10 [1.00, 1.17]	1.00 [1.00, 1.12]	1.10 [1.00, 1.17]	0.214
Fibrinogen (g/L)	3.00 [2.56, 3.50]	3.00 [2.60, 3.40]	3.16 [2.53, 3.76]	0.219
D-dimer (mg/L)	0.30 [0.20, 0.80]	0.30 [0.20, 0.52]	0.62 [0.22, 1.50]	<0.001
WBC (10^−9^/L)	7.30 [6.10, 8.60]	6.90 [6.00, 8.12]	8.05 [6.50, 9.78]	<0.001
Hemoglobin (g/L)	140.00 [130.00, 152.00]	141.00 [131.00, 152.00]	138.50 [125.25, 154.50]	0.658
Platelet (10^−9^/L)	215.00 [179.50, 250.75]	217.00 [183.00, 250.00]	209.50 [169.25, 256.25]	0.772
Triglycerides (mmol/L)	2.80 [1.72, 3.70]	3.00 [2.00, 3.80]	2.25 [1.60, 3.00]	0.002
LDL-C (mmol/L)	1.30 [1.10, 1.78]	1.30 [1.10, 1.40]	1.60 [1.20, 2.27]	<0.001
Bun (mmol/L)	5.80 [4.90, 6.77]	5.90 [5.00, 6.78]	5.25 [4.23, 6.75]	0.008
Scr (umol/L)	77.60 [67.20, 89.00]	80.20 [70.48, 89.43]	72.45 [59.95, 87.28]	0.002
CRP (mg/L)	2.26 [0.90, 5.57]	1.70 [0.80, 3.50]	5.40 [2.12, 10.35]	<0.001
Uric acid (umol/L)	410.29 [326.15, 472.00]	414.55 [337.60, 462.62]	380.00 [283.25, 494.00]	0.156
Glucose (mmol/L)	6.30 [5.23, 7.50]	6.20 [5.10, 7.50]	6.40 [5.40, 7.68]	0.211

Continuous variables are summarized as mean ± SD if approximately normally distributed and as median *IQR* if skewed; categorical variables are presented as *n* (%). LAD, left atrial diameter; LVEF, left ventricular ejection fraction; hs-cTnT, high-sensitivity cardiac troponin T; NT-proBNP, N-terminal pro–B-type natriuretic peptide; INR, international normalized ratio; WBC, white blood cell count; LDL-C, low-density lipoprotein cholesterol; BUN, blood urea nitrogen; Scr, serum creatinine; CRP, C-reactive protein; AF, atrial fibrillation.

### Feature selection and predictor set construction

In the training set, continuous variables underwent Spearman correlation analysis for collinearity (|ρ| ≥ 0.80); no highly correlated pairs were identified, and all 19 variables were retained (Supplementary Figure S1). Two complementary feature selection methods were applied to the training set: Method A involved univariable logistic regression with a significance threshold of *P* < 0.05 [Supplementary Table S1, which details odds ratios (OR) and 95% CI for each variable], and Method B used LASSO regression with the λ.1se rule (Supplementary Figure S2). The intersection of selected features from Methods A and B yielded seven predictors: Age, CRP, E/e′ ratio, LVEF, NT-proBNP, Triglycerides, and WBC. Based on these predictors, three nested predictor sets were defined: Model A (Age, CRP, Triglycerides, and WBC), Model B [Model A plus cardiac biomarkers (NT-proBNP)], and Model C [Model B plus echocardiographic parameters (LVEF and E/e′ ratio)].The overall modeling workflow is summarized in Supplementary Table S2 and illustrated in Supplementary Figures S3, S4.

### Model derivation, internal performance, and hold-out testing

**Figure 2:**
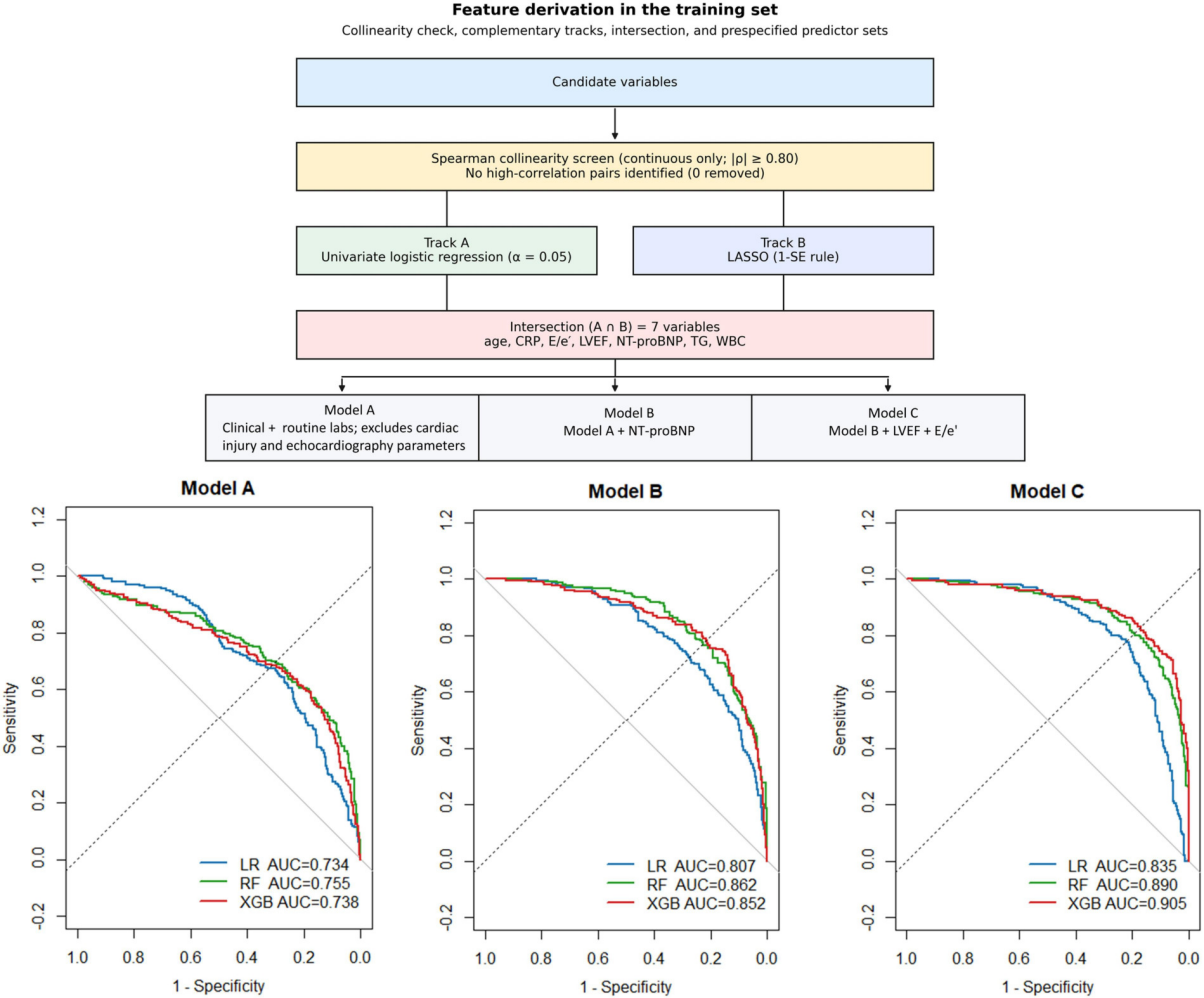
Workflow of variable selection and model development. The top panel illustrates the stepwise variable selection process used to derive three nested predictor sets: Model A (clinical and routine laboratory variables), Model B (Model A plus NT-proBNP), and Model C (Model B plus LVEF and the E/e′ ratio). The bottom panel presents receiver operating characteristic (ROC) curves for logistic regression (LR), random forest (RF), and extreme gradient boosting (XGB) models, evaluated on the training dataset across the three predictor sets. Area under the curve (AUC) values are shown within each plot. NT-proBNP, N-terminal pro–B-type natriuretic peptide; LVEF, left ventricular ejection fraction; E/e′, ratio of early mitral inflow velocity to mitral annular early diastolic velocity; TG, triglycerides; WBC, white blood cell count; AUC, area under the curve.

Across the training dataset, model discrimination improved progressively from Model A to Model B and further with Model C. Using Model A features alone, AUCs were modest (LR: 0.734, 95% CI: 0.692–0.775; RF: 0.755, 95% CI: 0.710–0.799; XGB: 0.738, 95% CI 0.693–0.784; Figure 2). Incorporating cardiac biomarkers (Model B) increased AUCs to 0.807–0.862 (Figure 2). Adding echocardiographic parameters, including LVEF and E/e′ (Model C), yielded the best training performance, with XGB achieving the highest AUC of 0.905 (95% CI: 0.877–0.933; threshold 0.310; sensitivity 0.831; specificity 0.832; precision 0.711; F1 score 0.766). LR and RF showed consistent increases in AUC (LR: 0.835, 95% CI: 0.802–0.868; RF: 0.890, 95% CI: 0.862–0.919). Thresholds were optimized by maximizing the Youden index for each model and predictor set.

In the test set, Model A features evaluated with XGB achieved an AUC of 0.856 (95% CI, 0.742–0.969; threshold 0.772; sensitivity 0.647; specificity 0.970; precision 0.917; F1 score 0.759). Incorporating cardiac biomarkers (Model B) improved performance across all algorithms [LR: 0.845 (95% CI, 0.737–0.953); RF: 0.891 (95% CI, 0.795–0.988); XGB: 0.914 (95% CI, 0.836–0.993)], with XGB demonstrating balanced sensitivity (0.941) and specificity (0.788). The combined Model C yielded the best discrimination, with RF and XGB achieving AUCs of 0.907 (95% CI, 0.829–0.986) and 0.906 (95% CI, 0.826–0.985), respectively, followed by LR (AUC 0.843, 95% CI, 0.730–0.956). Thresholds were optimized using the Youden index. Model performance metrics in both the training and test sets across nested predictor sets are summarized in Table 2.

**Table 2:** Scores for each model in the training and test sets.

Predictor set	ML	AUC (95% CI)	Threshold	Sensitivity	Specificity	Precision	F1
Training set							
Model A	LR	0.734 (0.692–0.775)	0.365	0.672	0.700	0.526	0.590
	RF	0.755 (0.710–0.799)	0.419	0.605	0.812	0.615	0.610
	XGB	0.738 (0.693–0.784)	0.427	0.595	0.819	0.620	0.607
Model B	LR	0.807 (0.772–0.843)	0.366	0.697	0.758	0.589	0.638
	RF	0.862 (0.832–0.893)	0.375	0.754	0.807	0.659	0.703
	XGB	0.852 (0.819–0.885)	0.373	0.744	0.850	0.711	0.727
Model C	LR	0.835 (0.802–0.868)	0.335	0.785	0.779	0.637	0.703
	RF	0.890 (0.862–0.919)	0.347	0.836	0.794	0.668	0.743
	XGB	0.905 (0.877–0.933)	0.310	0.831	0.832	0.711	0.766
Test sets							
Model A	LR	0.763 (0.621–0.905)	0.433	0.529	0.909	0.750	0.621
	RF	0.843 (0.730–0.956)	0.461	0.882	0.697	0.600	0.714
	XGB	0.856 (0.742–0.969)	0.772	0.647	0.970	0.917	0.759
Model B	LR	0.845 (0.737–0.953)	0.362	0.941	0.636	0.571	0.711
	RF	0.891 (0.795–0.988)	0.660	0.765	0.909	0.812	0.788
	XGB	0.914 (0.836–0.993)	0.393	0.941	0.788	0.696	0.800
Model C	LR	0.843 (0.730–0.956)	0.439	0.765	0.818	0.684	0.722
	RF	0.907 (0.829–0.986)	0.404	0.882	0.818	0.714	0.789
	XGB	0.906 (0.826–0.985)	0.488	0.882	0.788	0.682	0.769

Model A includes age, C-reactive protein (CRP), triglycerides, and white blood cell count (WBC); Model B incorporates Model A plus N-terminal pro-B-type natriuretic peptide (NT-proBNP); Model C comprises Model B plus left ventricular ejection fraction and E/e′ ratio. Operating characteristics were computed at the threshold maximizing the Youden index within the respective evaluation set and include sensitivity, specificity, precision value, and F1 score. ML, machine learning; LR, logistic regression; RF, random forest; XGB, extreme gradient boosting; AUC, area under the receiver operating characteristic curve; CI, confidence interval.

To evaluate clinical applicability, we constructed a simplified logistic regression model incorporating three key predictors (age, NT-proBNP, and E/e′), selected based on their clinical relevance and SHAP importance. This simplified model demonstrated good discrimination, with an AUC of 0.879 (95% CI, 0.821–0.937). However, its performance remained slightly inferior to that of the full machine learning models (Model C), in which RF and XGBoost achieved AUCs of 0.907 and 0.906, respectively.

### SHAP-based model interpretation

**Figure 3:**
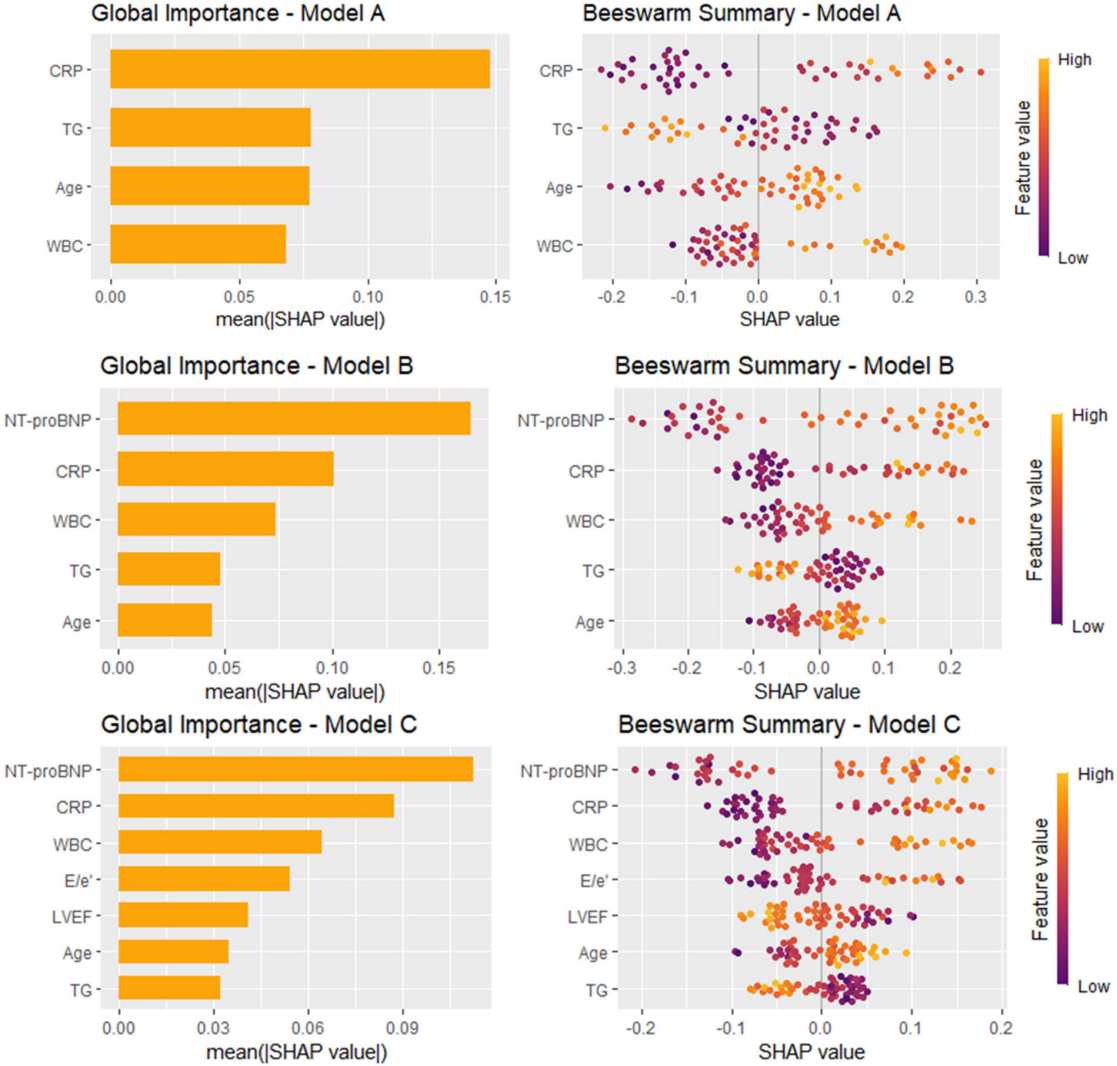
SHAP-based interpretation of the extreme gradient boosting models. Global importance plots (left panels) and beeswarm summary plots (right panels) illustrate the contribution of top-ranked features across the three predictor sets: Model A, Model B, and Model C. Bar charts show the mean absolute SHAP values, reflecting the average impact of each variable on model output. Beeswarm plots display the distribution of SHAP values for individual predictions, with color gradients indicating the raw feature values (purple: low, yellow: high). LVEF, left ventricular ejection fraction; NT-proBNP, N-terminal pro–B-type natriuretic peptide; WBC, white blood cell count; CRP, C-reactive protein.

XGB demonstrated consistently strong performance across Models A, B, and C in the test set, and was therefore selected for SHAP analysis. Global importance plots, based on mean absolute SHAP values, ranked CRP and triglycerides highest in Model A, followed by age and WBC count. In Model B, NT-proBNP emerged as the top predictor, with CRP and WBC next. Model C further highlighted NT-proBNP and CRP, alongside WBC, the E/e′ ratio, and LVEF. Beeswarm plots revealed directional trends across models: higher NT-proBNP and CRP levels were associated with increased predicted stroke risk (positive SHAP values), whereas higher LVEF was linked to reduced risk (negative SHAP values); E/e′ showed a positive association in Model C (Figure 3).

## Discussion

This study focuses on NVAF patients with CHA₂DS₂-VA scores ≤1, a clinically important subgroup in which stroke prevention strategies remain controversial. Although current guidelines generally classify these patients as low risk, our findings suggest substantial heterogeneity within this group. We deliberately focused on this population because patients with higher CHA₂DS₂-VA scores (≥2) already have clear guideline recommendations for anticoagulation, whereas decision-making in lower-risk patients remains more uncertain and therefore more clinically relevant. Identifying higher-risk individuals within this subgroup may have important implications for more personalized anticoagulation decision-making.

Although classified as low-to-intermediate risk by CHA2DS2-VA, patients within this stratum were not free of stroke, suggesting that clinical scores may be insufficient for individualized thromboembolic risk assessment. In contemporary cohorts, clinical scores provide only modest discrimination for thromboembolic events in AF, highlighting room for improvement (20). Risk assessment should therefore be individualized and informed by pathophysiological correlates—such as cardiac injury, hemodynamic stress, and prothrombotic tendency— in addition to traditional clinical factors (21, 22).

In this matched case-control cohort of patients with NVAF and CHA2DS2-VA scores ≤1, we identified a candidate predictor set through the intersection of two independent feature selection methods (univariable logistic regression and LASSO regression). We then applied a nested modeling strategy, progressing from Model A (clinical and routine laboratory variables) to Model B (Model A plus NT-proBNP) and Model C (Model B plus LVEF and E/e′), to evaluatethe incremental prognostic value of these biomarkers and echocardiographic parameters. This stepwise approach resulted in progressive improvement in discrimination, with AUCs increasing from 0.734–0.755 in Model A to 0.807–0.862 in Model B and 0.835–0.905 in Model C in the training set, and a test-set AUC of 0.906 with XGB for Model C.

These findings are consistent with and extend prior studies showing that stroke risk in atrial fibrillation may be underestimated when based solely on conventional clinical scores, particularly in patients considered low to moderate risk. Nishi et al. reported machine learning methods to atrial fibrillation cohorts for stroke prediction and demonstrated superior discrimination relative to traditional scoring (ROC-AUC 0.72, 95% CI: 0.66–0.79) compared with CHA₂DS₂-VASc (ROC-AUC 0.62, 95% CI: 0.54–0.70) (15). More broadly, recent studies have reportedAUCs ranging from 0.62 to 0.89 for machine-learning-based stroke prediction in AF (15, 23, 24). Against this background, our results suggest that combining biomarkers and echocardiographic markers with clinical variables may provide more refined risk stratification in NVAF patients with CHA₂DS₂-VA scores of 0–1. From a clinical perspective, the proposed model is not intended to replace existing risk scores, but rather to complement them by identifying higher-risk individuals within a group generally considered low risk.

The inherent “black-box” nature of machine learning models hinders the interpretation of individual feature contributions. To address this, we employed SHAP, a model-agnostic method that provides interpretable explanations by linking predictions to clinically relevant features. This approach enhances model transparency, facilitating explanations at the bedside (25). Our SHAP analyses revealed NT-proBNP and CRP as the most influential predictors of stroke risk in patients with NVAF and low CHA₂DS₂-VA scores, with consistently positive SHAP values signifying their association with heightened predicted probabilities. The stepwise addition of cardiac biomarkers in Model B substantially amplified NT-proBNP's contribution, highlighting its enhanced prognostic value relative to conventional clinical variables in Model A. Furthermore, echocardiographic parameters in Model C—most notably the E/e′ ratio (positive impact) and LVEF (protective effect)—refined risk discrimination, thereby emphasizing the role of diastolic dysfunction in subclinical thromboembolism among this ostensibly low-to-moderate-risk cohort. Previous studies have associated elevated NT-proBNP and CRP levels with increased ischemic stroke risk in NVAF populations (22, 26, 27). The ABC-Stroke score, incorporating NT-proBNP, corroborates this, with higher concentrations linked to elevated thromboembolic risk (28, 29). A substudy of ENGAGE AF-TIMI 48 further validated NT-proBNP's predictive utility for stroke in NVAF (28). CRP reflects systemic inflammation, promoting endothelial activation and a prothrombotic state (30), consistent with evidence that low-grade inflammation—indicated by elevated CRP and WBC counts—independently predicts recurrent cerebrovascular events and mortality (27, 31). In our cohort, stroke cases exhibited a higher inflammatory burden than controls (CRP: 5.40 [2.12–10.35] vs. 1.70 [0.80–3.50] mg/L; WBC: 8.05 [6.50–9.78] vs. 6.90 [6.00–8.12] × 10⁹/L), with laboratory values obtained from the initial blood draw upon presentation, as pre-stroke baselines were unavailable. These differences may partly reflect post-stroke inflammation, given sample collection following the index event. Additionally, stroke cases showed a higher E/e′ ratio [11.90 [10.40–14.10] vs. 9.60 [7.68–11.07]] and lower LVEF [61.00% (55.25–65.30) vs. 66.00% (62.00–69.25)]. The E/e′ ratio, a marker of elevated left ventricular filling pressures, has been independently linked to left atrial appendage thrombus in atrial fibrillation (AUC 0.83; adjusted odds ratio 1.13 per unit increase, 95% CI: 1.06–1.20) (32). Reduced LVEF, associated with atrial stasis, was incorporated in the externally validated CLOTS-AF model, achieving an AUC of 0.780 (95% CI: 0.707–0.853), surpassing CHA₂DS₂-VASc (AUC 0.619, 95% CI: 0.562–0.676) (33). This stratified approach, enriched with cardiac biomarkers and echocardiographic metrics, enhances risk assessment precision for low- to moderate-risk NVAF patients.

We implemented a prespecified, stability-focused analytical pipeline to ensure the reliability of conclusions. First, candidate features were screened using two parallel approaches: univariable logistic regression with a predefined significance threshold and LASSO regularization employing the *λ*.1se rule. This strategy enhances model stability and parsimony by retaining only predictors consistently significant across both methods, thereby minimizing spurious associations, multicollinearity, and overfitting. Second, three complementary machine learning algorithms (logistic regression, random forest, and extreme gradient boosting) were trained within a nested, stratified cross-validation framework (outer 5-fold, inner 3-fold) to decouple hyperparameter tuning from performance estimation and minimize overfitting. This approach leverages the complementary strengths of different modeling strategies and may enhance the generalizability and reliability of stroke risk stratification in nonvalvular atrial fibrillation patients. Performance metrics were aggregated across outer folds, followed by model refitting on the full training set and validation on an independent hold-out test set. Operating thresholds were determined by maximizing the Youden index. Finally, SHAP analyses provided model-agnostic interpretability, attributing predictions to clinically relevant features. This approach prioritizes reproducibility, generalizability, and interpretability over optimization of a single model.

However, this study has several limitations. First, as a single-center matched case–control study, it does not permit estimation of absolute risks or incidence, and selection bias cannot be excluded. Additionally, the single-center design and relatively small sample size may limit the generalizability of the findings. Second, biomarkers and echocardiography were single time-points; Anticoagulation exposure was not fully accounted for, allowing residual confounding. Third, we did not include left atrial strain or left atrial appendage anatomy/function, which may further improve discrimination and calibration. Future studies incorporating speckle-tracking left atrial strain and left atrial appendage morphology or flow parameters may further improve model performance. Finally, external validation in multi-center prospective cohorts is required before clinical application.

## Conclusion

In this study, we developed and internally validated machine-learning models to refine thromboembolic risk stratification in NVAF patients with CHA₂DS₂-VA scores ≤1. Models incorporating accessible cardiac biomarkers (NT-proBNP) and echocardiographic indices (E/e′, LVEF) demonstrated improved discrimination compared with clinical variables alone. These findings support more individualized risk assessment in this clinically uncertain population and warrant prospective, multi-center external validation before routine clinical implementation.

## Data Availability

The data analyzed in this study is subject to the following licenses/restrictions: The datasets generated and/or analyzed during the current study are not publicly available due to institutional policies involving patient privacy and data governance, but will be made available by the corresponding author upon reasonable request and subject to a data use agreement and applicable regulations. Requests to access these datasets should be directed to Feng Gao, silence4566@xmu.edu.cn.
