# Supplementary material for: Development and validation of interpretable machine learning models for predicting stroke in NVAF patients with CHA2DS2-VA scores ≤1
Source: Front Cardiovasc Med. 2026 Apr 28;13:1725168. doi: 10.3389/fcvm.2026.1725168 (PMC13160744; doi:10.3389/fcvm.2026.1725168)
Supplement: Supplementary file 1 [file Datasheet1.docx]

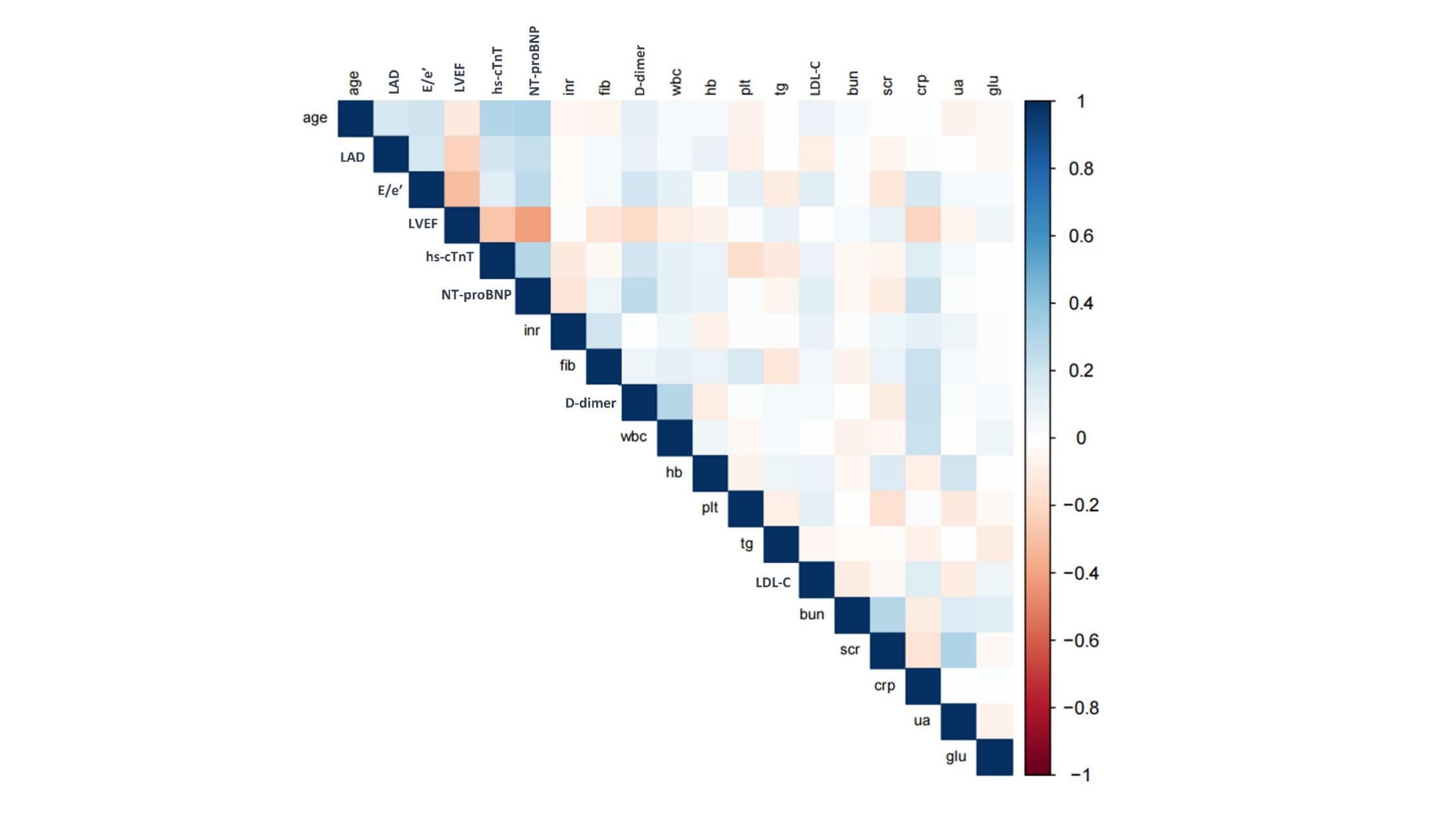


**Supplementary Figure S1. Pairwise correlation heatmap and collinearity screen.**
Lower-triangular heatmap of Spearman correlation coefficients among 19 continuous predictors (blue = positive, red = negative). A pre-specified collinearity threshold of |ρ| ≥ 0.80 was applied; no variable pairs exceeded the threshold, and consequently no variables were removed from subsequent modeling. Abbreviations: LAD, left atrial diameter; E/e′, early transmitral flow velocity to mitral annular velocity ratio; LVEF, left ventricular ejection fraction; hs-cTnT, high-sensitivity cardiac troponin T; NT-proBNP, N-terminal pro-B-type natriuretic peptide; INR, international normalized ratio; Fib, fibrinogen; WBC, white blood cells; Hb, hemoglobin; Plt, platelet; TG, triglycerides; LDL-C, low-density lipoprotein cholesterol; BUN, blood urea nitrogen; Scr, serum creatinine; CRP, C-reactive protein; UA, uric acid; Glu, glucose.


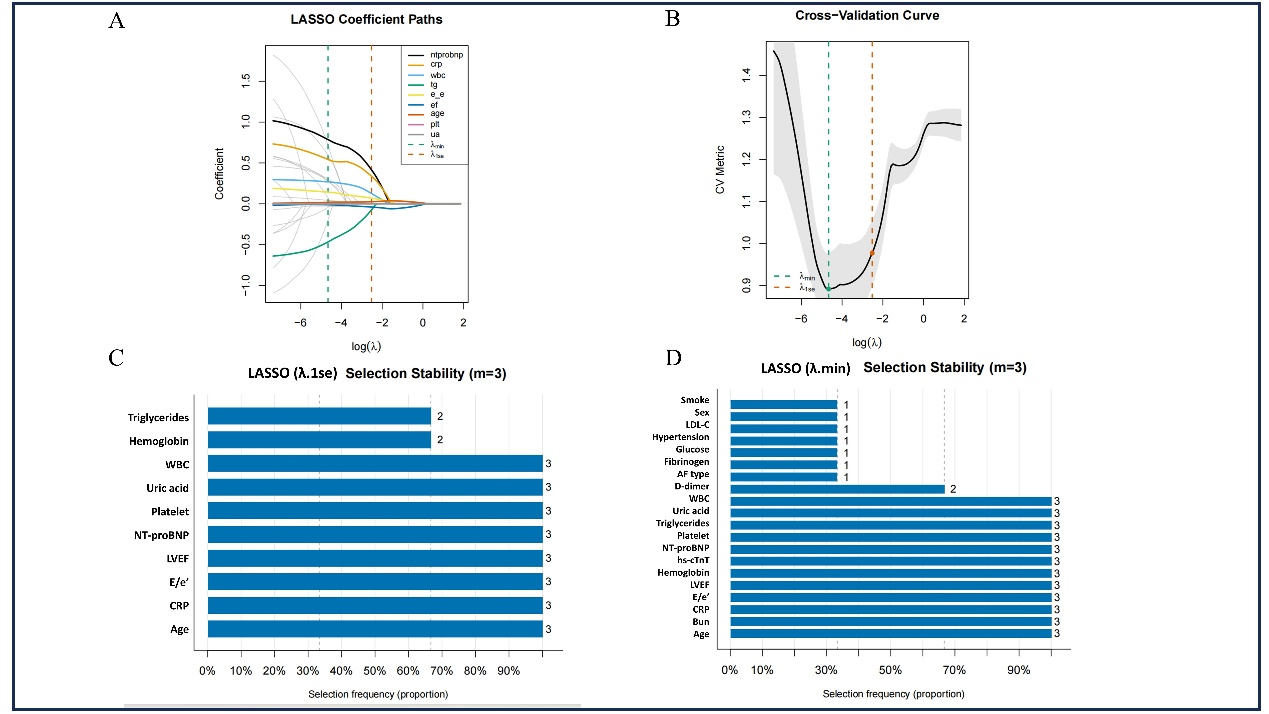


**Supplementary Figure S2. Variable selection via LASSO.**

Selection frequencies of variables derived from 5-fold cross-validated LASSO using the one-standard-error (1-SE) rule (λ.1SE) across m = 3 imputed datasets. Variables retained by majority vote (selection frequency ≥ 2/3) are highlighted. LVEF, left ventricular ejection fraction; hs-cTnT, high-sensitivity cardiac troponin T; NT-proBNP, N-terminal pro–B-type natriuretic peptide; WBC, white blood cell count; LDL-C, low-density lipoprotein cholesterol; BUN, blood urea nitrogen; CRP, C-reactive protein; E/e′, ratio of early mitral inflow velocity to mitral annular early diastolic velocity; AF, atrial fibrillation.


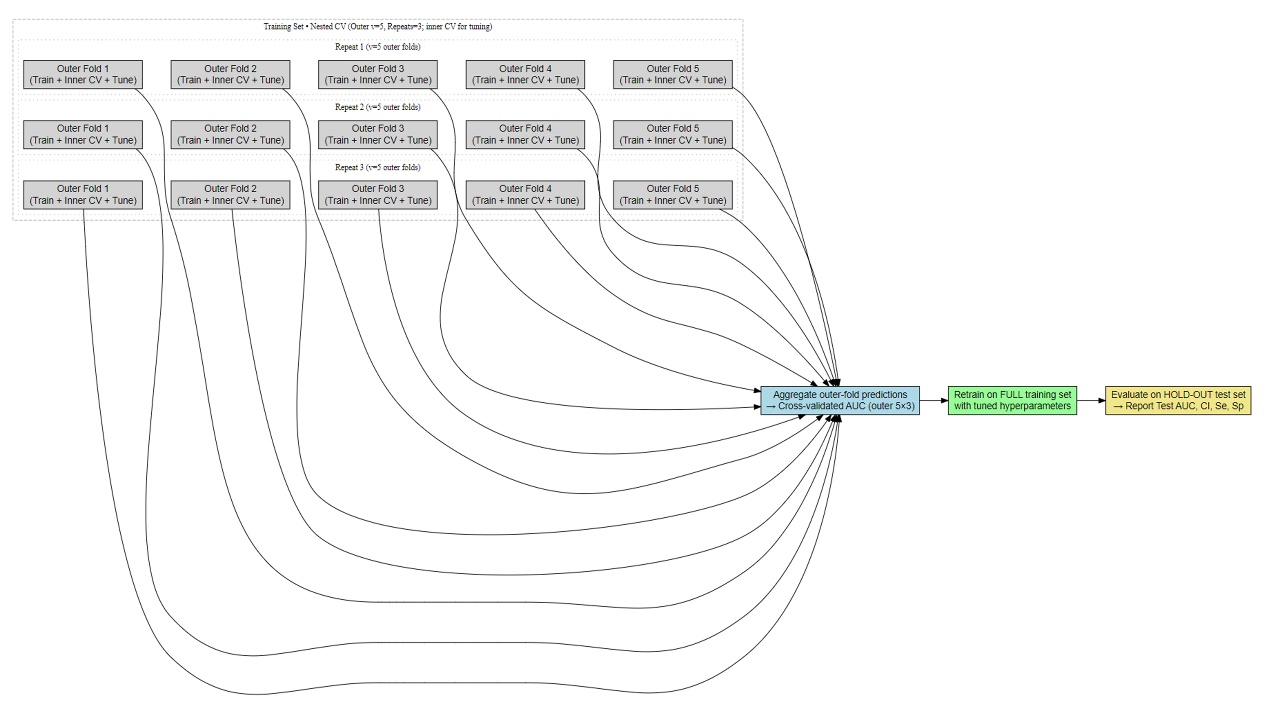


**Supplementary Figure S3. Workflow of model development and evaluation.**

Overview of the end-to-end pipeline. Nested cross-validation was used on the training set to obtain an unbiased estimate of model performance. After hyper-parameter tuning, the final model was refit on the full training set and then evaluated on the independent test set.


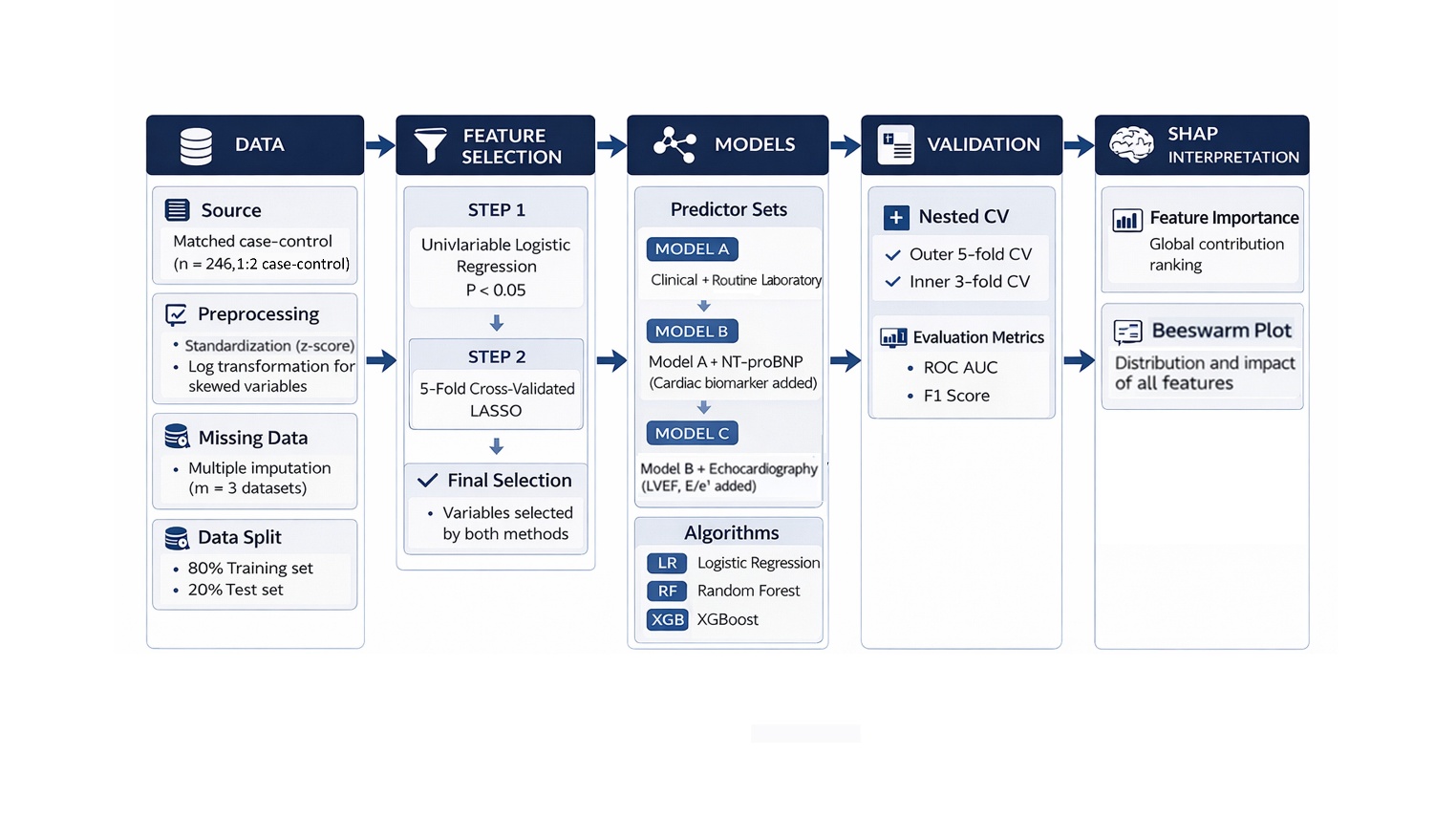


**Supplementary Figure S4. Overview of the machine learning modeling pipeline for stroke prediction in non-valvular atrial fibrillation (NVAF) patients with CHA₂DS₂-VA ≤1.**

Abbreviations: NT-proBNP, N-terminal pro–B-type natriuretic peptide; LVEF, left ventricular ejection fraction; E/e′, ratio of early mitral inflow velocity to mitral annular early diastolic velocity; LR, logistic regression; RF, random forest; XGB, extreme gradient boosting; LASSO, least absolute shrinkage and selection operator; ROC, receiver operating characteristic; AUC, area under the curve; CV, cross validation; SHAP, Shapley additive explanations.

Supplementary Table S1. Univariable logistic regression for candidate predictors

| Variable | OR (95%CI) | P value |
| --- | --- | --- |
| log_NT-proBNP | 3.614 (2.485–5.256) | <0.001 |
| log_CRP | 2.246 (1.704–2.960) | <0.001 |
| E/e' | 1.333 (1.203–1.478) | <0.001 |
| log_D-dimer | 2.053 (1.514–2.782) | <0.001 |
| LVEF (%) | 0.925 (0.892–0.958) | <0.001 |
| CHA2DS2-VA | 3.656 (2.011–6.645) | <0.001 |
| log_hs-cTnT | 2.315 (1.502–3.566) | <0.001 |
| WBC (10^9/L) | 1.309 (1.132–1.515) | <0.001 |
| Age (years) | 1.054 (1.024–1.084) | <0.001 |
| LDL-C (mmol/L) | 2.242 (1.363–3.686) | 0.001 |
| log-Scr | 0.174 (0.058–0.522) | 0.002 |
| Triglycerides (mmol/L) | 0.679 (0.531–0.868) | 0.002 |
| log-LAD | 12.888 (2.121–78.327) | 0.005 |
| HF (%) | 4.932 (1.471–16.536) | 0.010 |
| Hypertension (%) | 2.074 (1.126–3.820) | 0.019 |
| Age65_74 (%) | 2.110 (1.087–4.097) | 0.027 |
| AF_type | 1.586 (0.923–2.726) | 0.095 |
| Fibrinogen (g/L) | 1.322 (0.938–1.862) | 0.110 |
| Bun (mmol/L) | 0.883 (0.748–1.044) | 0.145 |
| Smoke (%) | 1.482 (0.870–2.526) | 0.148 |
| Vascular disease (%) | 0.474 (0.153–1.468) | 0.196 |
| log-INR | 0.453 (0.106–1.946) | 0.287 |
| Uric acid (umol/L) | 0.999 (0.997–1.001) | 0.291 |
| Glucose (mmol/L) | 1.056 (0.944–1.181) | 0.343 |
| Platelet (10^9/L) | 1.002 (0.998–1.006) | 0.405 |
| Hemoglobin (g/L) | 0.996 (0.982–1.011) | 0.624 |
| Alcohol (%) | 1.116 (0.584–2.133) | 0.739 |
| Diabetes mellitus (%) | 1.208 (0.281–5.182) | 0.800 |
| Male (%) | 1.000 (0.572–1.749) | 1.000 |

Odds ratios (ORs) with 95% confidence intervals (CIs) and two-sided PPP values are reported for each predictor. NT-proBNP, CRP, D-dimer, hs-cTnT, Scr, LAD, and INR were log-transformed due to marked skewness prior to analysis. Abbreviations: LAD, left atrial diameter; LVEF, left ventricular ejection fraction; hs-cTnT, high-sensitivity cardiac troponin T; NT-proBNP, N-terminal pro–B-type natriuretic peptide; INR, international normalized ratio; WBC, white blood cell count; LDL-C, low-density lipoprotein cholesterol; BUN, blood urea nitrogen; AF, atrial fibrillation.

**Supplementary Table S2. Overview of the machine learning modeling pipeline**

| **Step** | **Component** | **Description** | **Purpose** |
| --- | --- | --- | --- |
| **1** | Feature selection – Step 1 | Univariable logistic regression (significance threshold P < 0.05) | Initial screening of candidate predictors |
| **2** | Feature selection – Step 2 | LASSO regression (5-fold cross-validation) | Reduce multicollinearity and enhance model generalizability |
| 3 | Final predictor selection | Variables selected by both univariable logistic regression and LASSO methods | Enhance robustness and ensure stable variable selection |
| 4 | Predictor sets | Model A: clinical + routine laboratory variables; Model B: Model A + NT-proBNP;  Model C: Model B + echocardiographic parameters (LVEF, E/e′) | Evaluate incremental predictive value of progressively enriched predictor sets |
| 5 | Algorithms | Logistic regression (LR), Random forest (RF), XGBoost (XGB) | Compare performance between traditional statistical and machine learning models |
| 6 | Model validation | Nested cross-validation (outer 5-fold CV and inner 3-fold CV) | Optimize hyperparameters and reduce overfitting |
| 7 | Model evaluation | ROC curve, AUC, F1 score | Assess discrimination and classification balance |
| 8 | Model interpretation | SHAP (feature importance, beeswarm plots) | Improve interpretability of machine learning models |
